# Burden of Pulmonary Arterial Hypertension (PAH) on Patients Admitted for Asthma: A Nationwide Analysis, 2016-2020

**DOI:** 10.7759/cureus.100327

**Published:** 2025-12-29

**Authors:** Christian Siochi, Vaishvik K Patel, Aressa Canuto Miller, Farishta Ali, Patricia Guia Simoza, Daniela Bravo Solarte, Stephen Jesmajian

**Affiliations:** 1 Internal Medicine, Montefiore New Rochelle Hospital, Albert Einstein College of Medicine, New Rochelle, USA; 2 Internal Medicine, University of Connecticut School of Medicine, Farmington, USA; 3 Internal Medicine, Montefiore Einstein, New York City, USA

**Keywords:** asthma, critical care cardiology, pulmonary arterial pressure, pulmonary critical care, pulmonary hypertenion

## Abstract

Background

Pulmonary arterial hypertension (PAH) is known to impact other pulmonary disease outcomes, but there is a lack of data showing the degree of its impact. This study aims to elucidate the burden that PAH brings to patients admitted due to asthma.

Methods

The National Inpatient Sample (NIS) Database 2016-2020 was used to identify patients admitted due to asthma exacerbation. Patients admitted with a primary diagnosis of asthma, with or without a secondary diagnosis of PAH, were identified using International Classification of Diseases, 10th Edition (ICD-10) codes. PAH was classified using ICD-10 codes, specifically Group 3 PAH. The primary outcome was all-cause in-hospital mortality. Secondary outcomes were length of stay, resource utilization, and the necessity for endotracheal intubation. STATA v.13 (StataCorp LLC, College Station, TX, USA) was used for univariate and multivariate analysis. Data were considered statistically significant at p < 0.05.

Results

From 2016 to 2020, 491,990 patients were admitted due to asthma, and 7,860 had PAH. Patients with asthma and PAH had a 226% higher chance of dying during the index admission (OR 2.26; 95% CI 1.27-4.03; p = 0.006), an additional 0.75 days of hospital stay (regression coefficient 0.75; 95% CI 0.54-0.95; p < 0.001), and an average of $11,558.41 more spent per hospital stay (regression coefficient 11,558.41; 95% CI 8,657.33-14,459.49; p < 0.001). These patients had no statistically significant difference in endotracheal intubation rates (OR 1.69; 95% CI 0.80-1.94; p = 0.42).

Discussion

Asthma patients with PAH experience higher all-cause in-hospital mortality compared to those with asthma alone. These results highlight the increased risk associated with the coexistence of PAH and asthma, potentially due to the added hemodynamic burden imposed by PAH, which exacerbates respiratory and cardiovascular compromise. Activation of the transcription factor nuclear factor of activated T cells (NFAT) is an important marker known to link PAH and asthma. Due to its activation in both diseases, it may serve as a critical pathway for developing more effective therapeutic strategies targeting both conditions. There is also some research suggesting that increased neutrophils in asthma are correlated with PAH. Some limitations of using the NIS database are that it is subject to coding errors, misclassification, and variability across institutions, potentially affecting diagnostic and procedural accuracy.

Conclusion

PAH significantly impacts in-hospital outcomes in patients admitted due to asthma; thus, physicians should be aware of this difference. Future studies are needed to differentiate PAH patients by severity and to verify whether PAH treatment reverses this impact.

## Introduction

Asthma is a chronic inflammatory airway disease characterized by bronchial hyperresponsiveness and reversible airflow obstruction, affecting an estimated 260 million individuals worldwide and accounting for approximately 420,000 deaths annually [1]. Exposure to allergens, respiratory infections, tobacco smoke, exercise, cold air, and genetic predisposition contributes to airway inflammation and hyperresponsiveness. Persistent inflammation can lead to airway remodeling, including smooth muscle hypertrophy, subepithelial fibrosis, and increased bronchial vascularity, with asthmatic airways demonstrating higher vascular density and enlarged vessels compared with non-asthmatic airways [2]. These vascular changes contribute to airflow limitation, mucosal edema, and impaired gas exchange, particularly during severe exacerbations.

Pulmonary arterial hypertension (PAH) is a progressive and life-threatening disorder characterized by pathological remodeling of the pulmonary microvasculature, resulting in elevated pulmonary vascular resistance and eventual right ventricular failure [3]. In the United States, PAH remains a rare condition, affecting approximately 1 in 94,000 individuals, yet it is associated with substantial morbidity and mortality despite advances in therapy [4]. Hallmark pathological features include smooth muscle proliferation, endothelial dysfunction, inflammation, and in situ thrombosis, leading to luminal narrowing and increased right ventricular afterload [5].

Although PAH is classified into five groups based on etiology, Group 3 PAH, which arises from chronic lung diseases and hypoxemia, is particularly relevant to asthma [6]. Chronic airway inflammation, hypoxic vasoconstriction, and vascular remodeling in asthma may predispose susceptible patients to pulmonary vascular dysfunction, suggesting a pathophysiological continuum between severe asthma and PAH. Emerging evidence indicates shared mechanisms between asthma and PAH, including inflammatory signaling, smooth muscle hyperplasia, endothelial dysfunction, and angioproliferative remodeling [7]. These overlapping processes may amplify cardiopulmonary stress during acute asthma exacerbations, particularly in the inpatient setting.

The coexistence of PAH in patients with asthma may therefore have important clinical implications. Elevated pulmonary pressures and right ventricular dysfunction can impair pulmonary perfusion, worsen ventilation-perfusion mismatch, and limit physiologic reserve during asthma exacerbations. This may translate into higher in-hospital mortality, prolonged hospitalization, and increased resource utilization. Despite these plausible mechanisms, data examining the impact of PAH on acute inpatient outcomes among asthma hospitalizations remain limited, with existing studies largely focused on outpatient disease burden, physiologic overlap, or small-cohort analyses.

Inpatient outcomes were selected as the focus of this study because hospitalization for asthma represents a period of heightened vulnerability, where cardiopulmonary interactions are most likely to influence clinical outcomes and healthcare utilization. Large administrative databases allow for the evaluation of rare but high-risk comorbidities, such as PAH, at a national level, providing insight into short-term outcomes that are not easily captured in smaller prospective cohorts.

This study aims to evaluate the association between PAH and in-hospital outcomes among patients admitted with asthma, including mortality, length of stay, and healthcare costs, using a nationally representative inpatient database. By focusing on acute hospitalizations, this analysis seeks to address an important gap in the literature and clarify whether PAH identifies a higher-risk asthma population during inpatient care.

This work was previously presented at the ATS 2024 Conference on May 19, 2024.

## Materials and methods

Study design

This was a retrospective cohort study using discharge data from the National Inpatient Sample (NIS), the Healthcare Cost and Utilization Project (HCUP), and the Agency for Healthcare Research and Quality, from 2016 to 2020 [8].

Study inclusion criteria

Patients with a non-elective admission primary diagnosis of asthma exacerbation, aged >18 years, with or without PAH, were identified using International Classification of Diseases, 10th Edition, Clinical Modification (ICD-10-CM) and Procedure Coding System (ICD-10-PCS) codes. The ICD-10 codes used to classify asthma include J45.2 (mild intermittent asthma), J45.3 (mild persistent asthma), J45.4 (moderate persistent asthma), J45.5 (severe persistent asthma), and J45.9 (unspecified asthma). PAH is classified under codes I27.0 (primary pulmonary hypertension) and I27.20 (unspecified pulmonary hypertension) [9]. Asthma exacerbation was identified using the primary diagnosis only. Duplicate hospitalizations were not excluded, and all etiologies of PAH were included.

Ethical considerations

The data from the NIS-HCUP are publicly available, de-identified, and exempt from institutional review board approval. The need for informed consent was waived.

Outcome measures

The primary outcome of interest is all-cause in-hospital mortality, while secondary outcomes include length of stay, total hospital charges, and rate of endotracheal intubation.

Statistical analysis

This study used a confidence interval (CI) of 95% and a p-value <0.05 as statistically significant in its analysis. Continuous variables were examined through the calculation of means accompanied by standard deviations, or medians along with interquartile ranges in the case of normally distributed and skewed data, respectively. Descriptive statistics, incorporating frequencies and percentages, were employed for the analysis of categorical variables. Patient- and hospital-level baseline characteristics and in-hospital outcomes were compared between patients with a primary diagnosis of asthma exacerbation, aged >18 years, with or without a secondary diagnosis of PAH, using the Pearson χ² test for categorical variables and the independent sample t-test for continuous variables. To calculate unadjusted and adjusted odds ratios for in-hospital clinical outcomes, univariate and multivariate logistic regression were used. To account for potential confounding factors, a multivariate regression model was adjusted for patient- and hospital-level baseline characteristics with a p-value <0.2. All analyses were conducted using STATA v.13 (StataCorp LLC, College Station, TX, USA).

## Results

Baseline patient characteristics, including race and Charlson comorbidity index, and hospital-level characteristics are shown in Table 1 for patients admitted with a primary diagnosis of asthma exacerbation, with or without a secondary diagnosis of PAH. The Charlson comorbidity index was controlled for in the adjusted model, as described in the Methods section/Table 1. In the United States, between 2016 and 2020, of the 491,990 patients who had a primary diagnosis of asthma, 7,860 had PAH and 484,130 did not. The asthma population with PAH had a mean age of 63.58 years, while those without PAH had a mean age of 50.60 years.

**Table 1:** Percentage of patients with a primary diagnosis of asthma, stratified by baseline patient- and hospital-level characteristics, with or without a secondary diagnosis of PAH. To account for potential confounding factors, a multivariate regression model was later adjusted for patient- and hospital-level baseline characteristics, with a p-value <0.2. PAH: Pulmonary arterial hypertension

Baseline characteristics of asthma patients		% of asthma patients without PAH (N = 484,130)	% of asthma patients with PAH (N = 7,860)	% of total asthma patients (N = 491,990)	p-value
Sex	Male	27.41	18.83	27.27	<0.01
	Female	72.59	81.17	72.73	
Race	White patients	44.41	43.27	44.39	<0.01
	Black patients	32.34	35.06	32.38	
	Hispanic patients	16.28	13.45	16.24	
	Asian patients	2.71	4.14	2.73	
	Native American patients	0.68	0.84	0.68	
	Other	3.58	3.23	3.58	
Charlson comorbidity index	0	0	0	0	<0.01
	1	61.04	20.74	60.39	
	2	21.56	27.99	21.67	
	>3	17.4	51.27	17.94	
Annual median income ($)	<49,999	37.35	34.89	37.31	0.136
	50,000-64,999	24.71	26.85	24.75	
	65,000-85,999	21.56	21.14	21.55	
	>86,000	16.38	17.12	16.39	
Insurance type	Medicare	33.77	61.25	34.22	<0.01
	Medicaid	29.94	18.96	29.77	
	Private insurance	27.1	17.09	26.93	
	Self pay	9.19	2.71	9.08	
Hospital region	Northeast	26.29	24.81	26.27	0.359
	Midwest	19.47	21.25	19.5	
	South	37.47	37.34	37.47	
	West	16.77	16.6	16.77	
Hospital bed size	Small	24.7	21.63	24.65	0.02
	Medium	31.93	32	31.93	
	Large	43.37	46.37	43.42	
Hospital location	Rural	8.1	5.42	8.06	<0.01
	Urban	91.9	94.58	91.94	
Hospital teaching status	No	31.23	24.35	31.13	<0.01
	Yes	68.77	75.65	68.87	

The majority of patients included in this study were white (44.39%, N = 218,394.36), female (72.73%, N = 357,824.33), and had a Charlson comorbidity index of 1 (60.39%, N = 297,112.76). Tables 1 detail the percentage of patients with a primary diagnosis of asthma exacerbation according to baseline patient and hospital-level characteristics, with or without a secondary diagnosis of PAH.

Primary outcomes

Unadjusted outcomes showed that all-cause in-hospital mortality was significantly higher in asthma with PAH patients compared to asthma without PAH patients (OR 2.33; 95% CI 1.31-4.14; p = 0.004) (Table 2).

**Table 2:** Unadjusted odds ratio for the all-cause in-hospital mortality outcome of patients admitted for asthma with a secondary diagnosis of PAH. This study used a 95% CI and a p-value <0.05 as statistically significant in its analysis. PAH: Pulmonary arterial hypertension

Unadjusted primary outcome						
Outcome	Odds ratio	Std error	t	p-value	95% CI	
All-cause inpatient mortality	2.33	0.68	2.87	0.004	1.31	4.14

Adjusted outcomes showed that asthma with PAH patients had a 226% higher chance of all-cause in-hospital mortality during index admission (OR 2.26; 95% CI 1.27-4.03; p = 0.006) (Table 3).

**Table 3:** Adjusted odds ratio for the in-hospital mortality outcome of patients admitted for asthma with a secondary diagnosis of PAH. This study used a 95% CI and a p-value <0.05 as statistically significant in its analysis. PAH: Pulmonary arterial hypertension

Primary outcome adjusted for variables with p < 0.2						
Outcome	Odds ratio	Std error	t	p-value	95% CI	
All-cause inpatient mortality	2.26	0.67	2.77	0.006	1.27	4.03

Secondary outcomes 

As shown in Table 4, unadjusted outcomes revealed that asthma with PAH patients averaged an additional 1.33 days longer during hospital stay (regression 1.33; 95% CI 1.15-1.51; p < 0.001) and $17,014.01 more dollars spent for the hospital stay (regression 17,014.01; 95% CI 14,271.92-19,756.10; p < 0.001).

**Table 4:** Unadjusted regression coefficients for the length of stay and total hospital charges outcomes of patients admitted for asthma with a secondary diagnosis of PAH. This study used a 95% CI and a p-value <0.05 as statistically significant in its analysis. PAH: Pulmonary arterial hypertension; LOS: Length of stay in days; TOTCHG: Total hospital charges in dollars

Unadjusted secondary outcomes						
Outcome	Regression	Std error	t	p-value	95% CI	
LOS	1.33	0.09	14.3	<0.001	1.15	1.51
TOTCHG ($)	17,014.01	1398.96	12.16	<0.001	14,271.92	19,756.10

As shown in Table 5, adjusted outcomes revealed that asthma with PAH patients averaged an additional 0.75 days longer during hospital stay (regression 0.75; 95% CI 0.54-0.95; p < 0.001) and $11,558.41 more dollars spent for the hospital stay (regression 11,558.41; 95% CI 8,657.33-14,459.49; p < 0.001).

**Table 5:** Adjusted regression coefficient for length of stay and total hospital charges outcomes of patients admitted for asthma, with a secondary diagnosis of PAH. This study used a 95% CI and a p-value <0.05 as statistically significant in its analysis. PAH: Pulmonary arterial hypertension; LOS: Length of stay in days; TOTCHG: Total hospital charges in dollars

Secondary outcomes adjusted for variables with p < 0.2						
Outcome	Regression	Std error	t	p-value	95% CI	
LOS	0.75	0.11	7.04	<0.001	0.54	0.95
TOTCHG ($)	11,558.41	1480.08	7.81	<0.001	8,657.33	14,459.49

Unadjusted outcomes showed that the need for endotracheal intubation was not significantly different between asthma patients with PAH and asthma patients without PAH (OR 0.85; 95% CI 0.58-1.26; p = 0.42) (Table 6).

**Table 6:** Unadjusted odds ratio for endotracheal intubation outcomes of patients admitted for asthma, with a secondary diagnosis of PAH. This study used a 95% CI and a p-value <0.05 as statistically significant in its analysis. PAH: Pulmonary arterial hypertension; ETT: Endotracheal intubation

Unadjusted additional secondary outcome						
Outcome	Odds ratio	Std error	t	p-value	95% CI	
ETT	0.85	0.17	-0.8	0.42	0.58	1.26

Adjusted outcomes showed that the need for endotracheal intubation was not significantly different between asthma patients with PAH and asthma patients without PAH (OR 1.692; 95% CI 0.8-1.94; p = 0.34) (Table 7).

**Table 7:** Adjusted odds ratio for the endotracheal intubation outcomes of patients admitted for asthma, with a secondary diagnosis of PAH. This study used a 95% CI and a p-value <0.05 as statistically significant in its analysis. PAH: Pulmonary arterial hypertension; ETT: Endotracheal intubation

Additional secondary outcome adjusted for variables with p < 0.2						
Outcome	Odds ratio	Std error	t	p-value	95% CI	
ETT	1.24	0.28	0.95	0.34	0.8	1.94

## Discussion

The findings of this study demonstrate a significant association, rather than a causal relationship, which supports the finding that asthma patients with PAH experience significantly higher all-cause in-hospital mortality compared to those with asthma alone. This highlights the increased risk associated with the coexistence of PAH and asthma, potentially due to the added hemodynamic burden imposed by PAH, which exacerbates respiratory and cardiovascular compromise. As mentioned by Said et al., asthma and PAH share important pathological features, including inflammation, smooth muscle contraction, and remodeling [10]. In our adjusted analyses, the association between PAH and mortality remained significant even after accounting for these comorbid conditions, suggesting an independent contribution of PAH to inpatient risk. Although these features are often manifested unequally in the two disorders, they can be explained by a shared pathogenetic mechanism: activation of nuclear factor of activated T cells (NFAT) [10].

NFAT is a regulator of inducible gene transcription that is activated during the immune response. NFAT activation promotes smooth muscle cell proliferation and contributes to the proliferative reaction in airway remodeling. These findings are based on experimental models showing that airway remodeling is accompanied by remodeling of smaller pulmonary arteries, validating the hypothesis of a similar pathogenesis in asthma and PAH. Furthermore, lungs with increased NFAT expression in mice exhibited airway hyperresponsiveness, airway inflammation, and PAH with vascular remodeling, all of which were reversible with NFAT treatment [8]. This was also supported by Rosival, who stated that hyperventilation with hypocapnia in patients with bronchial asthma can be sufficiently explained by hypertension in the pulmonary artery, which is always registered when the pulmonary artery has been catheterized in such patients [11].

Vasoactive intestinal peptide (VIP) in aerosol has shown airway-relaxant, anti-inflammatory, and anti-proliferative actions. VIP seems to be emerging as a likely physiological inhibitor of the NFAT pathway, which explains why deletion of the VIP gene results in the expression of both asthma and PAH phenotypes. VIP could become a future therapeutic modulator to mitigate PAH in patients with asthma [10]. Future research into the NFAT pathway may offer novel insights into the overlapping mechanisms driving both asthma and PAH, providing a foundation for more effective therapeutic strategies targeting both conditions. Knowing that NFAT is a molecule responsible for inflammatory and remodeling effects, it is important to investigate whether commonly used steroids in asthma, such as inhaled corticosteroids, exert any regulatory influence on NFAT activity. A detailed understanding of this interaction may reveal whether these steroids can attenuate NFAT-driven pathways and ultimately reduce the incidence or progression of PAH in asthma patients [12]. While experimental models implicate NFAT activation and VIP dysregulation as shared contributors to airway and vascular remodeling, these findings must be interpreted cautiously, as animal and preclinical data may not fully translate to human disease.

The relationship between asthma and PAH is an emerging area of research, with limited studies directly examining mortality rates in patients diagnosed with both conditions. The increased length of hospital stays and total hospital charges observed in our investigation of asthma patients with PAH further highlight the complex nature of managing these patients. Unadjusted outcomes revealed an additional 1.33 days of hospitalization, which remained significant at 0.75 days after adjusting for confounders. This suggests that PAH contributes to prolonged hospital stays, even after accounting for other clinical variables. Possible explanations for this finding include the relationship between similar comorbidities in patients with asthma and PAH, as described by Aboelnasr et al. in their study, where they noted a significant increase in the prevalence of diabetes, obesity, and heart failure in the asthma and PAH group. This suggests that the cardiometabolic phenotype increases the risk for multifactorial mechanisms [13].

Pulmonary hypertension is independently associated with higher in-hospital mortality, invasive mechanical ventilation, and increased healthcare utilization in asthma exacerbation hospitalizations [13]. These processes not only contribute to prolonged hospital stays but also to increased healthcare utilization and adverse asthma exacerbation outcomes. Attentive care is essential in this population to optimize asthma control, manage comorbidities, and potentially slow the progression of PAH, ultimately improving patient outcomes and resource utilization.

Asthma is a lung condition associated with type 3 pulmonary hypertension. A case report by Kawashima et al. suggests that patients experiencing exacerbations of asthma and COPD may benefit from surgeries such as tracheal stenosis repair and diaphragmatic eventration. These interventions resulted in a 23% improvement in forced vital capacity and a 56% improvement in forced expiratory volume in one second. Additionally, transthoracic echocardiography demonstrated a +12% improvement in left ventricular ejection fraction and a reduction of 20 mmHg in right ventricular systolic pressure [14]. These findings are relevant in the context of asthma patients with coexisting PAH, as they suggest that addressing structural abnormalities contributing to respiratory compromise could offer an alternative therapeutic approach to managing PAH. In patients where pharmacologic modulation of pathways like NFAT or VIP is insufficient or contraindicated, such surgical interventions may represent a viable option to improve both pulmonary and cardiac function.

In contrast to the increase in mortality, total hospital charges, and hospital length of stay, the need for endotracheal intubation was not significantly different between asthma patients with and without PAH. This finding may suggest that PAH does not independently influence the severity of respiratory failure requiring mechanical ventilation. However, given the variability in clinical practice patterns, thresholds for intubation, and patient factors such as comorbidities and baseline functional status, this result should be interpreted with caution. Additional studies examining the interplay between PAH, asthma exacerbations, and mechanical ventilation needs are warranted to confirm these findings and identify potential nuances.

There is limited research directly linking increased mortality in patients with both asthma and PAH, as observed in our study. However, other studies have clearly demonstrated an association between cardiovascular comorbidities, pulmonary hypertension, and asthma [13]. In one such publication, conditions like hypertensive cardiomyopathy and pulmonary hypertension were found to be significantly associated with asthma, with hypertensive cardiomyopathy showing a very strong association, and pulmonary hypertension demonstrating a strong association [15]. While causal inferences cannot be made, early recognition of PAH, careful volume and oxygenation management, and multidisciplinary collaboration involving pulmonology and cardiology may be beneficial in optimizing inpatient care.

Limitations

This study relies on the NIS, an administrative database that lacks the clinical granularity of electronic health records. As such, important confounders could not be accounted for, including pulmonary function test results, asthma severity, PAH severity or hemodynamic parameters, functional class, duration of disease, medication use (such as inhaled corticosteroids, biologics, vasodilator therapies, or diuretics), smoking status, and adherence to treatment. Diagnostic and procedural coding inaccuracies or misclassification may also influence the validity of the findings. Given the retrospective, observational design, temporal or causal relationships cannot be inferred, and the observed associations may be affected by residual and unmeasured confounding.

Additionally, the use of inpatient-only data introduces the possibility of selection bias, as the analysis is limited to hospitalized patients and may preferentially capture individuals with more severe disease. The NIS does not include outpatient encounters, longitudinal follow-up, or post-discharge outcomes, precluding assessment of readmissions, long-term mortality, or disease progression. Consequently, the impact of PAH on asthma outcomes outside the index hospitalization cannot be evaluated. While the findings are derived from a large, nationally representative U.S. inpatient sample, generalizability to non-U.S. healthcare systems may be limited due to differences in healthcare access, treatment practices, and patient demographics.

Despite these limitations, the study highlights a clinically meaningful association between PAH and worse inpatient outcomes among asthma patients, including increased mortality, prolonged hospitalization, and higher healthcare costs. These findings suggest that PAH may identify a higher-risk subgroup within hospitalized asthma patients, rather than establish a direct causal relationship. The observed economic burden further underscores the potential value of targeted risk stratification and resource planning. Future prospective studies, incorporating detailed clinical data, are needed to validate these results, elucidate underlying mechanisms, and determine whether stratification by PAH severity, etiology, or treatment status modifies asthma-related outcomes. Such studies may also clarify whether integrated pharmacologic or multidisciplinary management strategies could mitigate the adverse outcomes observed in this population.

## Conclusions

This study highlights the significant association between PAH and adverse outcomes among patients hospitalized for asthma. Our findings demonstrate that asthma patients with concomitant PAH experience higher in-hospital mortality, longer lengths of stay, and increased healthcare costs compared with those without PAH. While rates of endotracheal intubation were not significantly different, the overall inpatient burden associated with PAH in this population remains substantial. Importantly, these findings represent associations rather than causal relationships, given the retrospective and observational nature of the analysis. From a clinical perspective, the observed associations suggest that the presence of PAH may serve as a marker of higher-risk asthma hospitalizations, warranting heightened awareness and careful inpatient management, rather than specific therapeutic recommendations beyond the evidence presented.

Future studies, incorporating prospective designs and granular clinical data, are needed to validate these findings and to better characterize disease severity. In particular, stratification by PAH severity, hemodynamic parameters, or etiologic subtype may clarify whether certain PAH phenotypes disproportionately contribute to worse asthma-related outcomes and could inform more targeted risk stratification in future analyses.
